# The follicle-stimulating hormone triggers rapid changes in mitochondrial structure and function in porcine cumulus cells

**DOI:** 10.1038/s41598-023-50586-3

**Published:** 2024-01-03

**Authors:** Amel Lounas, Yann Breton, Ariane Lebrun, Isabelle Laflamme, Nathalie Vernoux, Julie Savage, Marie-Ève Tremblay, Martin Pelletier, Marc Germain, François J. Richard

**Affiliations:** 1 Centre de recherche en reproduction, développement et santé intergénérationnelle (CRDSI), Département des sciences animales, Faculté des Sciences de l’agriculture et de l’alimentation, Université Laval, Québec, QC G1V 0A6 Canada; 2 Centre de recherche du CHU de Québec-Université Laval, Axe Maladies infectieuses et immunitaires, Département de microbiologie-infectiologie et d’immunologie, Faculté de médecine, Université Laval, Québec, QC G1V4G2 Canada; 3 Centre de recherche du CHU de Québec-Université Laval, Axe Neurosciences, Département de médecine moléculaire, Université Laval, Québec, QC G1V 4G2 Canada; 4 Division of Medical Sciences, University of Victoria, Victoria, BC V8W 2Y2 Canada; 5 Département de biologie médicale, Université du Québec à Trois-Rivières, Québec, G8Z 4M3 Canada

**Keywords:** Hormone receptors, Reproductive biology

## Abstract

Oocyte maturation is a key process during which the female germ cell undergoes resumption of meiosis and completes its preparation for embryonic development including cytoplasmic and epigenetic maturation. The cumulus cells directly surrounding the oocyte are involved in this process by transferring essential metabolites, such as pyruvate, to the oocyte. This process is controlled by cyclic adenosine monophosphate (cAMP)-dependent mechanisms recruited downstream of follicle-stimulating hormone (FSH) signaling in cumulus cells. As mitochondria have a critical but poorly understood contribution to this process, we defined the effects of FSH and high cAMP concentrations on mitochondrial dynamics and function in porcine cumulus cells. During in vitro maturation (IVM) of cumulus-oocyte complexes (COCs), we observed an FSH-dependent mitochondrial elongation shortly after stimulation that led to mitochondrial fragmentation 24 h later. Importantly, mitochondrial elongation was accompanied by decreased mitochondrial activity and a switch to glycolysis. During a pre-IVM culture step increasing intracellular cAMP, mitochondrial fragmentation was prevented. Altogether, the results demonstrate that FSH triggers rapid changes in mitochondrial structure and function in COCs involving cAMP.

## Introduction

Oocyte maturation is an important process during which the female germ cell undergoes meiotic resumption and completes its preparation prior to fertilization including cytoplasmic molecular maturation, cytoplasmic organelle maturation, and epigenetic maturation. In ovarian follicles, immature oocytes are arrested at the first prophase of the meiotic division, which needs to be re-initiated for oocytes to complete their development and transition into competent mature gametes^1^. To achieve this maturation, the oocyte is supported by its somatic environment, including surrounding cumulus cells that communicate closely with the female germ cell. Within the COCs, nutrients and energy substrates such as pyruvate and lactate transit from the cumulus cells to the oocyte through gap junctions, which provide the oocyte the energy source required for its maturation, fertilization, and embryo developement^2,3^. The critical role of cumulus cells on the oocyte is supported by results showing impaired nuclear maturation and developmental competence when removed before the end of IVM^4,5^.

Given the crucial role of mitochondria in adenosine triphosphate (ATP) production, mainly through oxidative phosphorylation that relies on the tricarboxylic acid (TCA) cycle to generate the necessary reducing equivalents nicotinamide adenine dinucleotide (NADH2) and flavin adenine dinucleotide (FADH2) from different metabolic substrates and a wide range of metabolic processes, mitochondria likely play an important role in cumulus-oocyte communication. In fact, mitochondrial dysfunction in cumulus cells affects oocyte maturation and developmental competence^6^. Mitochondrial activity is regulated by changes in organelle structure, including fission and fusion^7^. These changes in mitochondrial morphology occur within minutes to hours in response to metabolic signals. They allow to adjust energy demands and are controlled by a family of large dynamin-related GTPases^8^. Mitochondrial fission is mediated by Dynamin-related Protein-1 (DRP1), while Mitofusins (Mfns) 1 and 2 and Optic atrophy type 1 (OPA1) are required for mitochondrial fusion^9–11^. Importantly, alterations in mitochondrial dynamics modulated by fusion and fission processes impair proper cellular responses^12^. For example, the inhibition of DRP1 activity during IVM of porcine COC induced mitochondrial dysfunction, resulting in failed oocyte nuclear maturation and cumulus cell expansion^13^.

FSH plays a central role in follicle growth and oocyte development and is required for follicle survival, cell differentiation, and proliferation^14^. It is well known that FSH modulates the acquisition of oocyte competence development, steroid production, and cumulus expansion in both in vivo and in vitro maturation conditions^15–17^. Classical IVM media are often supplemented with growth factors and hormones, such as FSH and luteinizing hormone (LH), to promote successful oocyte maturation^18,19^. FSH is commonly added to IVM media as it improves oocyte quality and developmental competence^20,21^. However, oocytes themselves are deficient in their ability to respond to FSH and require the presence of cumulus cells to transduce FSH signaling^22,23^.

FSH transduces its signal through a transient spike in cAMP synthesis by the adenylyl cyclase enzyme in the ovarian follicle’s somatic cells^24^. The surge of cAMP following FSH stimulation is a key regulator of oocyte meiotic resumption, with high levels of cAMP being inhibitory^25^. Modulation of intracellular cAMP levels during IVM is thus used as a strategy to improve oocyte quality and their ability to develop into embryos by inhibiting the resumption of meiosis, thereby helping to maintain communication and exchange between cumulus cells and the oocyte. As reviewed, the development of two-stage IVM systems, including a pre-IVM culture step (with cAMP modulators) followed by a standard IVM culture, illustrates the gain in oocyte quality^24^.

**Figure 1:**
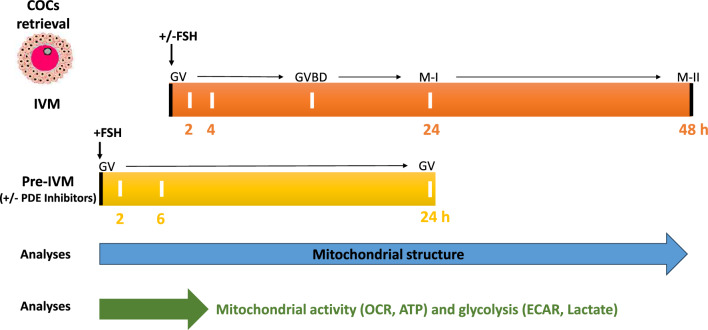
Scheme of the experimental design showing both IVM and pre-IVM culture steps, and the different selected culture times as well as the carried-out treatments and analysis. COCs: cumulus-oocyte complexes, GV: germinal vesical, GVBD: germinal vesicle breakdown, M-I: metaphase I, M-II: metaphase II, IVM: in vitro maturation + / − FSH, Pre-IVM: pre-IVM culture step + FSH and + / − phosphodiesterases inhibitors (to block oocyte maturation at GV stage) or Forskolin, TMRM (Tetramethylrhodamine, Methyl Ester, Perchlorate), SEM (scanning electron microscopy), OCR: oxygen consumption rate, ECAR: extracellular acidification rate.

We previously described mitochondrial morphology in porcine cumulus cells, showing that these cells display complex mitochondrial networks^26^. Here, we defined the effect of IVM on mitochondrial morphology and activity (ATP levels, oxygen consumption) in porcine cumulus cells (Fig. 1). We specifically addressed the role of FSH, a key signal for oocyte maturation, and its downstream effector cAMP. We found that exposure of cumulus cells to FSH during IVM promotes a transient increase in mitochondrial length. This increase is followed by mitochondrial fragmentation, which is prevented by maintaining elevated cAMP levels. Surprisingly, the short-term mitochondrial elongation observed upon FSH stimulation was associated with a decrease in mitochondrial activity. Our study thus demonstrates that FSH regulates mitochondrial structure and function during IVM. As pre-IVM culture using high cAMP improves IVM^24^, our study suggests that modulating mitochondrial activity could similarly lead to improved IVM.

## Results

### Changes in mitochondrial dynamic during IVM

**Figure 2:**
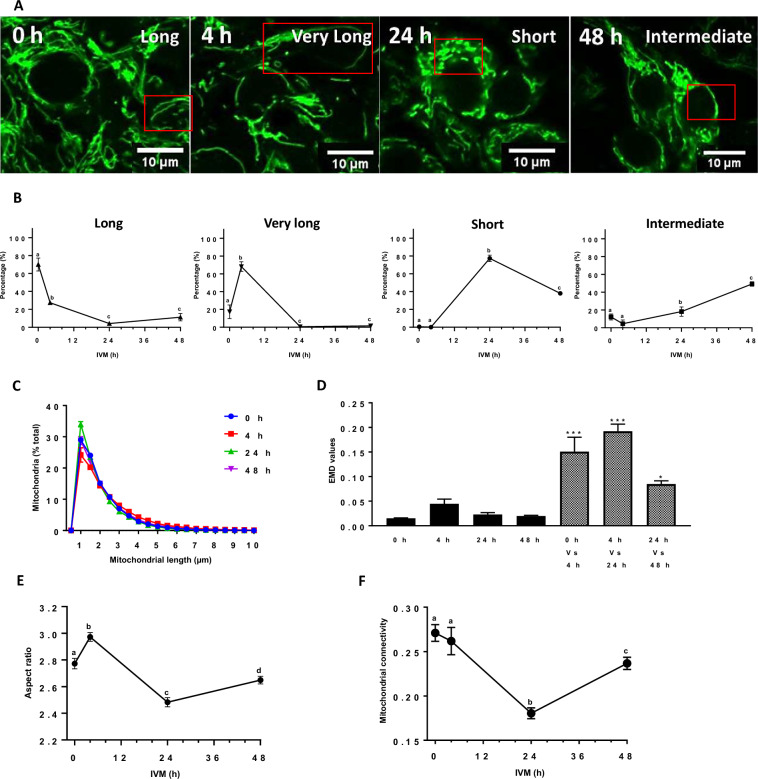
Mitochondrial dynamics in cumulus cells for different IVM periods (0, 4, 24, and 48 h). (**A**) Representative images of TMRM-stained cumulus cell mitochondria observed live by confocal microscopy at 63X after different IVM periods (0, 4, 24, and 48 h). (**B**) Manual quantification of mitochondrial network phenotypes reported as a percentage in each cumulus cell from 10 COCs for different IVM periods (0, 4, 24, and 48 h). Mitochondria were manually classified as very long, long, intermediate, and short, depending on their length in each cumulus cell. (**C**) Quantification of mitochondrial length distribution with the Momito algorithm for each IVM period. (**D**) EMD quantification of the differences between mitochondrial length distribution for different IVM periods. For each condition, EMD values were first calculated between each cell within a distribution type, and the average for that distribution to calculate the experimental variation for each IVM period presented as black histograms. Striped histograms refer to the EMD between distribution types (0 h Vs. 4 h, 4 h Vs. 24 h, 24 h Vs. 48 h). Each cell within a distribution type was compared to the average for each of the other distributions. (**E**) Mitochondrial aspect ratio and (**F**) Mitochondrial connectivity changes during IVM. All data are expressed as the average of three independent experiments ± SEM (10 COCs per replicate for each IVM time). (* *p* < 0.05; *** *p* < 0.001). Different letters indicate statistically significant differences (*p* < 0.05).

To determine the effect of IVM on mitochondrial morphology in porcine cumulus cells, we isolated COCs (10 COCs by replicate for each culture condition, n = 3) from antral follicles, stained their active mitochondria with Tetramethylrhodamine, Methyl Ester, Perchlorate (TMRM, 150 nM^26,27^), and imaged them at different IVM times (0, 4, 24, and 48 h). Observation of live cumulus cells by confocal microscopy and the manual classification of each cell (120 cumulus cells by 10 COCs per replicate for each culture condition, n = 3) depending on its mitochondrial phenotype, as described^28^, revealed major changes in organelle morphology during IVM (Fig. 2A,B). At 0 h, long mitochondria were abundant (70%), and after 4 h, most mitochondria were very long (73%). Most mitochondria had changed structure and size to short forms after 24 h of IVM and intermediate ones at 48 h.

We then performed a quantitative analysis of mitochondrial length using the Momito algorithm^28^. Consistent with the manual classification, this analysis revealed alterations in mitochondrial length across the examined IVM times (Fig. 2C). Interestingly, mitochondria longer than 2 µm increased from 44% ± 0.38 to 55% ± 2.09 after 4 h of IVM (*p* = 0.001, t-test), while those with a 1 µm length increased from 24% ± 1.74 to 34% ± 0.56 after 24 h (*p* = 0.002, t-test), suggesting an elongation of mitochondria followed by their fragmentation after 4 h and 24 h of IVM, respectively.

To further define the changes in mitochondrial length distribution during IVM, we calculated the differences between the mitochondrial distribution for each time point using the Earthmover distance (EMD), which measures the difference between distributions (see methods for details in the calculation)^28^. We first calculated EMD scores for individual distributions relative to the average distribution of the same time point, which represents the experimental variability (Fig. 2D, black bars). To measure the difference between distributions, we then calculated EMD values between time points (Fig. 2D, grey bars). Statistical analysis of EMD values indicated a significant difference in the mitochondrial length distribution. Similarly, mitochondrial aspect ratio, which measures the length-to-width ratio of mitochondria, transiently increased at 4 h and then significantly decreased, consistent with an elongation of mitochondria followed by their fragmentation (Fig. 2E). Mitochondrial shortening at 24 h IVM was also observed as a decrease in mitochondrial connectivity (mitochondrial junctions/mitochondrial ends) (Fig. 2F), connectivity being lower when mitochondria are fragmented^28^.

### FSH alters mitochondrial structure and function during IVM

**Figure 3:**
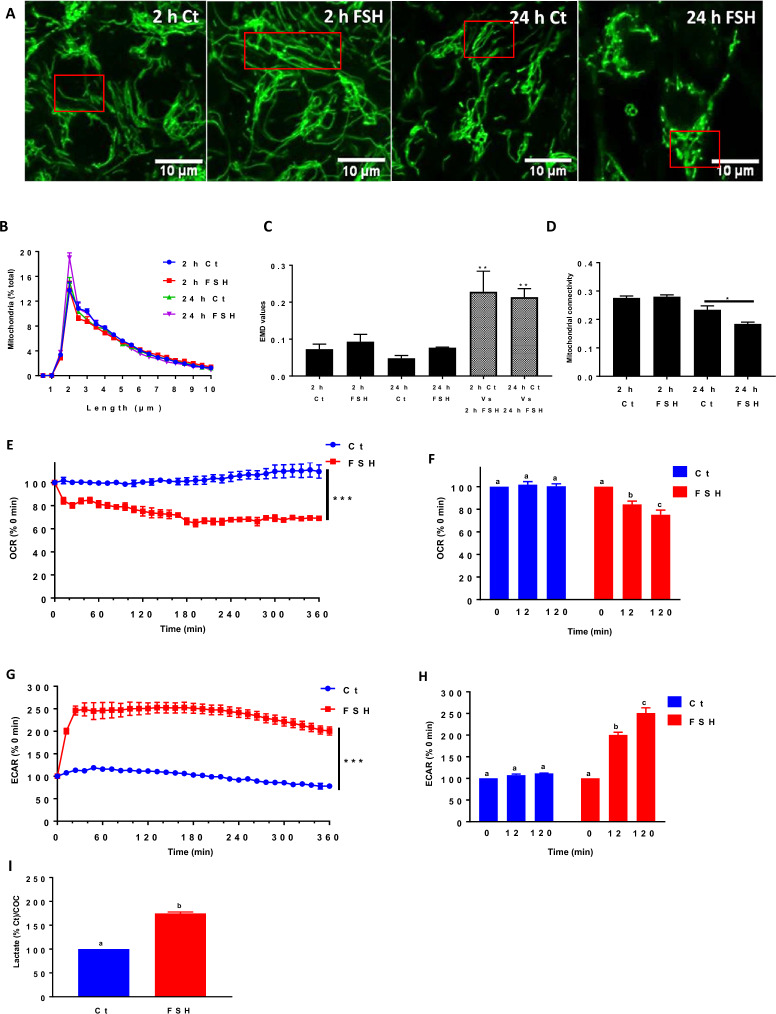
Effects of FSH on mitochondrial dynamics and activity in cumulus cells, as well as their metabolic status during IVM. (**A**) Representative images of TMRM-stained mitochondria in cumulus cells exposed to FSH or not (Ct) for 2 h and 24 h of IVM. (**B**) Momito analysis of mitochondrial length distribution for each IVM condition. (**C**) The calculated EMD values for each culture condition (black histograms represent experimental variability for each IVM time; striped histograms refer to the measured statistical difference between two length distributions). (**D**) Momito analysis of mitochondrial connectivity for each IVM condition. Data of TMRM staining are expressed as the average of three independent experiments ± SEM (10 COCs for each IVM time). (**E**) Real-time kinetics of oxygen consumption rates (OCR) measured in COC during IVM by seahorse analyzer in response to FSH relative to control, which was set at 100% at 0 h. (**F**) Changes in the relative basal respiration measured by seahorse analyzer in control COCs and COCs exposed to FSH for different IVM periods. (**G**) Real-time kinetics of relative extracellular acidification rates (ECAR) measured in COCs by seahorse analyzer during IVM after FSH injection compared to control, which was set at 100% at 0 h. (**H**) Changes in basal glycolysis percentage measured by seahorse analyzer in control COCs and COCs exposed to FSH for different IVM periods. All data of seahorse analysis are expressed as the average of four independent experiments ± SEM (20 COCs by well in triplicate). (**I**) Lactate production in COCs cultured 2 h with FSH compared to control. Data of lactate measurements are expressed as the average of three independent experiments ± SEM with 20 COCs by well in triplicate per replicate, and different letters indicate statistically significant differences (*p* < 0.05). (* *p* < 0.05; *** *p* < 0.001).

FSH is required for oocyte maturation and developmental competence within the ovarian follicule^14^. The supplementation of FSH in the IVM medium induces important metabolic changes in cumulus cells, including steroid synthesis, glucose uptake, and glycogen consumption^29,30^. To determine how FSH affects mitochondrial response, we first determined mitochondrial structure in COCs (10 COCs by replicate for each culture condition, n = 3) incubated for different times in the absence or presence of FSH (at 0.01 µg/ml which has been optimized for cumulus cells expansion and the percentage of meiotic resumption measured as germinal vesicle breakdown (GVBD))^31^. Mitochondria were longer in cumulus cells (120 cumulus cells by 10 COCs per replicate for each culture condition, n = 3) after 2 h of culture with FSH compared to incubation in the absence of the hormone, as mitochondria longer than 2 µm increased from 54% ± 0.54 to 65% ± 0.71 in the presence of FSH (*p* = 0.0001, t-test) (Fig. 3A,B), with the EMD values showing a significant difference in mitochondrial length distribution between the conditions (Fig. 3C). Furthermore, mitochondria became fragmented at 24 h only in the presence of FSH (mitochondria less than 2 µm in length increased from 29% ± 0.51 in control to 34% ± 0.48 with FSH, *p* = 0.0006, t-test) and showed a significant difference in mitochondrial length distribution compared to control (Fig. 3A–C). Consistent with this finding, there was a significant decrease in mitochondrial connectivity at 24 h only in the presence of FSH (Fig. 3D). Altogether, these results indicate that FSH triggers a change in mitochondrial dynamics during IVM.

Changes in mitochondrial dynamics impact mitochondrial activity^32^. To determine whether the mitochondrial changes we observed in cumulus cells upon exposure to FSH could increase mitochondrial activity, we measured oxygen consumption rates (OCR) of COCs (20 COCs/well in culture miniplates in triplicate, n = 4) in real-time during 6 h in the absence and the presence of FSH. A rapid and significant decrease in total OCR was observed in response to FSH compared to control (Fig. 3E). The reduction in OCR was observed as early as 12 min after FSH addition and was maintained for at least 360 min of culture (Fig. 3E,F). Simultaneously, the extracellular acidification rate (ECAR) increased rapidly and significantly in the presence of FSH compared to the control (Fig. 3G,H). As increased ECAR is often associated with elevated aerobic glycolysis and lactate production, we directly measured lactate produced in response to FSH^33^. As shown in Fig. 3I, the lactate level significantly increased (*p* < 0.0001) twofold compared to the control, suggesting an increase in glycolysis.

### FSH inhibits mitochondrial activity

**Figure 4:**
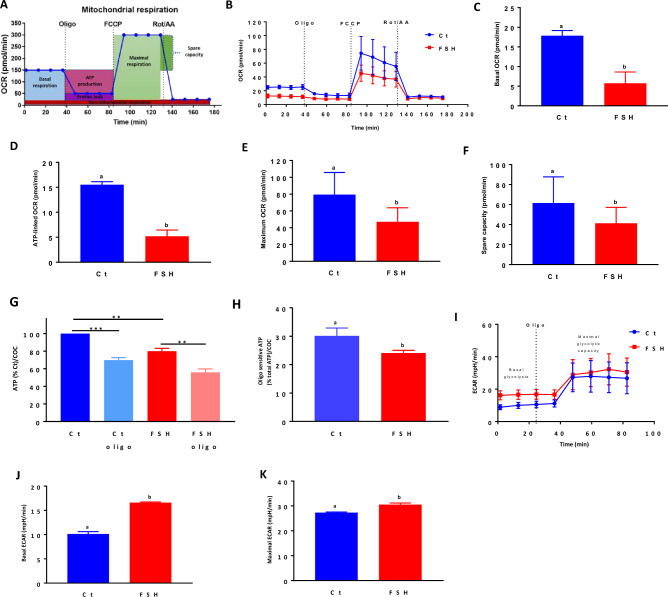
Mito stress test of mitochondrial respiration parameters as well as metabolic changes in COCs cultured 2 h with FSH compared to control. (**A**) An example of mitochondrial respiration pattern measured as oxygen consumption rate (OCR) according to the Agilent Seahorse Mito Stress cell protocol. (**B**) Mitochondrial respiration changes measured using a seahorse analyzer in control (Ct) COCs and COCs treated 2 h with FSH using mitochondrial drugs. (**C**) Basal respiration of COCs cultured 2 h in the presence and absence of FSH. (**D**) Oligomycin-sensitive ATP-linked mitochondrial respiration of COCs cultured 2 h in the presence and absence of FSH, (**E**) Mitochondrial maximal respiration of COCs cultured 2 h in the presence and absence of FSH, (**F**) Mitochondrial spare capacity of COCs cultured 2 h with FSH compared to control. (**G**) Percentage of ATP synthesis in COCs after 2 h of culture with FSH in the presence and absence of oligomycin (oligo). (**H**) Oligomycin-sensitive ATP-linked mitochondrial respiration percentage in COCs cultured with FSH for 2 h compared to control. (**I**) Extracellular acidification rate (ECAR) changes in control COCs and COCs exposed 2 h to FSH using the mitochondrial inhibitor oligomycin. (**J**) Basal glycolysis and (**K**) maximal glycolytic capacity of control COCs and COCs exposed 2 h to FSH. All seahorse data are expressed as the average of five independent experiments ± SEM (20 COCs by well in triplicate). Different letters indicate statistically significant differences by paired Student t-test (*p* < 0.05). Data of ATP measurements are expressed as the average of three independent experiments ± SEM with 20 COCs by well in triplicate per replicate (** *p* < 0.01, *** *p* < 0.001). Different letters indicate statistically significant differences (*p* < 0.05).

We then investigated the effect of a 2 h incubation with FSH on specific cellular energetic parameters, which were measured following the sequential administration of mitochondrial modulators (Fig. 4A,B). FSH significantly decreased the basal mitochondrial respiration (calculated after subtracting non-mitochondrial respiration) of COCs (20 COCs/well in culture miniplates in triplicate, n = 5) compared to the control (Fig. 4C). ATP-linked OCR and maximal respiration were also significantly lower following a 2 h culture in the presence of FSH (Fig. 4D,E), suggesting a decrease in the capacity of these mitochondria to produce ATP. The reduction in mitochondrial respiration after 2 h culture with FSH was also accompanied by a decrease in the mitochondrial spare capacity, which is the difference between basal respiration and mitochondrial maximal activity (Fig. 4F). In fact, a 2 h culture with FSH significantly decreased total ATP content (Fig. 4G), together with the fraction of total ATP sensitive to the ATP synthase inhibitor oligomycin (oligo at 10 µM^34^) (Fig. 4H). In addition, the 2 h FSH treatment caused an increase in basal and maximal ECAR (Fig. 4I–K), consistent with increased glycolysis.

**Figure 5:**
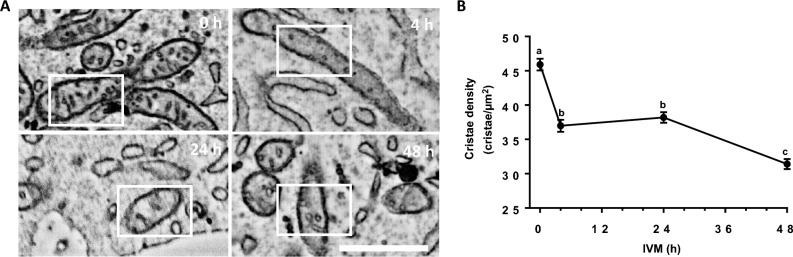
Mitochondrial ultrastructure analysis in cumulus cells in response to FSH during IVM. (**A**) Scanning electron microscopy micrographs showing mitochondrial cristae changes in response to FSH during different IVM times (0, 4, 24 and 48 h). (**B**) Cristae density for each culture period (0, 4, 24, and 48 h) (n = 16 cumulus cells for each IVM period, mean ± SEM). Different letters indicate statistically significant differences (*p* < 0.05).

Mitochondrial OCR depends on the electron transport chain that is assembled in mitochondrial cristae, the invaginations of the mitochondrial inner membrane^35^. The number of cristae within a mitochondrion thus has an important effect on its capacity to generate ATP^36,37^. As mitochondria from cells exposed to FSH for 2 h were less active despite being elongated, we measured mitochondrial cristae density in cumulus cells (450 mitochondria from 16 cells for each culture condition) exposed to FSH using scanning electron microscopy (SEM) analysis. While well-developed mitochondrial cristae were observed at each IVM time point (0, 4, 24, and 48 h) (Fig. 5A), their density dramatically decreased as early as 4 h after FSH stimulation (Fig. 5A,B), consistent with the observed decrease in both ATP production and mitochondrial basal respiration in response to FSH during IVM.

Altogether, our results indicate that although FSH promotes mitochondrial elongation in the time frame examined here (2 h), this leads to a sharp decrease in mitochondrial basal respiration, ATP synthesis, mitochondrial maximal respiration, and mitochondrial spare capacity, leading to a metabolic switch toward glycolysis. Our results thus support a diminution of mitochondrial activity by FSH.

### High-cAMP pre-IVM culture step delays mitochondrial fragmentation

**Figure 6:**
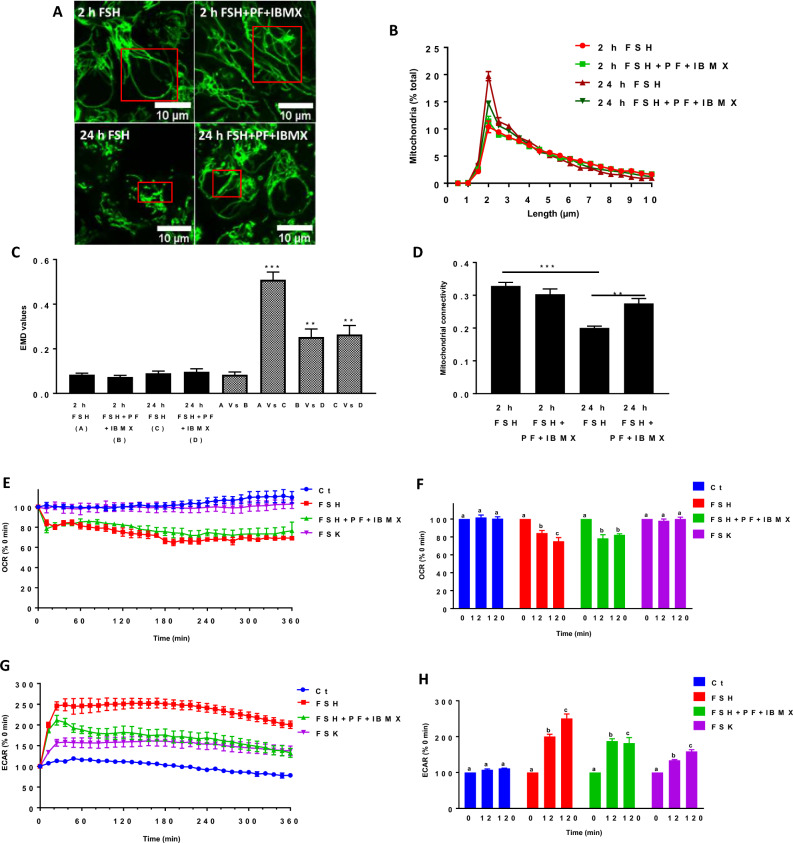
Effect of high cAMP pre-IVM culture step on FSH-mediated mitochondrial response in COCs. (**A**) Representative images of TMRM stained mitochondria in cumulus cells cultured for 2 h and 24 h with FSH combined or not to PDE inhibitors (PF-04957325 and IBMX). (**B**) Momito analysis of mitochondrial length distribution for each culture condition. (**C**) EMD value quantification showing the shift in mitochondrial length distribution between the different culture conditions (black histograms represent experimental variability for each culture time; striped histograms refer to the difference between two length distributions from different conditions). (**D**) Mitochondrial connectivity for each culture condition. All data of TMRM staining are expressed as the average of three independent experiments ± SEM (10 COC for each condition per replicate). (** *p* < 0.01; *** *p* < 0.001). (**E**) Kinetic of oxygen consumption rate (OCR) percentage of COCs in real-time during pre-IVM culture step after FSH, FSH + PDE inhibitors, and forskolin (FSK) injection compared to control. (**F**) Changes in basal respiration percentage of COCs exposed to FSH, FSH + PDE inhibitors, FSK, and control COCs for different culture periods. (**G**) Kinetic of extracellular acidification rate (ECAR) percentage of COCs in real-time during pre-IVM culture step after FSH, FSH + PDE inhibitors, and FSK injection compared to control. (**H**) Changes in basal glycolysis percentage of COCs after FSH, FSH + PDEs inhibitors, and FSK injection and control COC for different pre-IVM culture step periods. All data are expressed as the average of four independent experiments ± SEM (20 COC by well in duplicate). Different letters indicate statistically significant differences (*p* < 0.05).

High cAMP levels during pre-IVM impact oocyte quality and cellular processes such as meiotic resumption and cytoplasmic maturation^25,38^. It is also known that high cAMP concentrations within the COC maintain gap junction communication and nutrient transfer to the oocyte, resulting in oocyte quality improvement^2^. Here, we investigated the effects of a pre-IVM culture step using high cAMP levels, raised by specific phosphodiesterase (PDE) inhibitors (PF-04957325 (300 nM)^31^ + 3-Isobutyl-1-methylxanthine (IBMX, 500 µM)^39^), on FSH-dependent mitochondrial responses^40^. The morphology of active mitochondria was studied in cumulus cells (120 cumulus cells by 10 COCs per replicate for each culture condition, n = 3) using confocal images of TMRM-stained mitochondria after 2 and 24 h of culture with FSH, combined or not with PDE inhibitors. Cumulus mitochondria were elongated at 2 h following FSH addition, with most mitochondria being longer than 2 µm in the presence or absence of PDE inhibitors (67% ± 2.24 with FSH vs 65% ± 1.68 with inhibitors (*p* = 0.50, t-test)) (Fig. 6A,B) and the EMD between the two distributions being similar to the experimental variation of each time point (Fig. 6C). In contrast, PDE inhibitors prevented the mitochondrial fragmentation caused by a 24 h exposure to FSH (Fig. 6A), with the mitochondrial population under 2 µm of length decreasing in the presence of PDE inhibitors from 34% ± 1.70 to 28% ± 0.25 (*p* = 0.50, t-test) (Fig. 6B). Mitochondrial length distributions at 24 h were also significantly different as measured by their EMD values (Fig. 6C). In addition, the decrease in mitochondrial connectivity observed in cumulus cells exposed to FSH for 24 h was no longer observed following the administration of PDE inhibitors (Fig. 6D). Altogether, these results indicate that the high cAMP pre-IVM culture step using PDE inhibitors^40^ prevented the long-term FSH-induced mitochondrial fragmentation.

As we found that the changes in mitochondrial structure caused by FSH were associated with a decrease in mitochondrial activity, we next studied the effects of high cAMP pre-VM culture step on mitochondrial activity in COCs (20 COCs/well in culture miniplates in triplicate, n = 4). We measured OCR and ECAR in response to FSH in the presence and absence of PDE inhibitors. Consistent with the lack of effect produced by PDE inhibitors on mitochondrial structure at 2 h, FSH induced a rapid and significant decrease in the OCR after 12 min, with or without PDE inhibitors present (Fig. 6E and Fig. 1A in supplemental data). Interestingly, while ECAR increased rapidly and significantly in response to FSH alone, this response was partially prevented by the addition of PDE inhibitors (Fig. 6G and Fig. 1B in supplemental data), suggesting that the effect of FSH on OCR and ECAR are mechanistically distinct. Thus, we directly tested the effect of high levels of cAMP synthesis independently of FSH stimulation by treating COCs with forskolin (100 µM)^40^, an adenylyl cyclase activator^39^. In contrast to FSH, forskolin did not alter basal OCR (Fig. 6E,F). It did, however, cause a partial increase in ECAR (Fig. 6G,H), suggesting that the effect of FSH on OCR is not directly dependent on cAMP compared to ECAR.

A high concentration of cAMP improves oocyte quality by delaying oocyte meiotic resumption^41^. Our data suggest that high cAMP pre-IVM culture step impacts cumulus mitochondrial structures and functions.

## Discussion

FSH plays a crucial role in oocyte maturation^14^. Signaling downstream of the FSH receptor is mediated through a transient increase in cAMP that affects multiple cellular processes in the cumulus cells, forming an electrophysiological syncytium during oocyte nuclear maturation, steroidogenesis, and cumulus expansion^15–17^. Here, we show that FSH regulates mitochondrial structure in cumulus cells during IVM. Specifically, exposure of COCs to FSH causes a transient elongation of mitochondria, followed by mitochondrial shortening. These changes in mitochondrial morphology were accompanied by a rapid decrease in basal mitochondrial respiration and total ATP levels and an increase in glycolysis. Our results indicate that FSH regulates mitochondrial dynamics and activity in cumulus cells during IVM, leading to a sustained metabolic switch. Importantly, the high cAMP pre-IVM culture step delayed the long-term FSH-dependent changes.

Mitochondria are highly dynamic organelles that rapidly change their morphology and activity in response to cellular signaling through a process termed mitochondrial dynamics^42,43^. For example, changes in nutrient availability, including amino acid starvation, trigger mitochondrial elongation required to maintain cellular ATP. Interestingly, this occurs through cAMP-dependent inhibition of the fission protein DRP1. Mitochondrial morphology also changes during the cell cycle, where mitochondria elongate between the G1 and S stages, where more energy is required, and become fragmented at the G2 stage^42,44^. During IVM, cumulus cells not only undergo differentiation but also proliferation^45^. As cumulus cells proliferate in response to FSH, the mitochondrial morphology may follow the cell cycle^46^.

Mitochondrial elongation is thought to make mitochondria more efficient by promoting tight cristae structure and the diffusion of metabolites across the mitochondrial network. Nevertheless, we observed a rapid decrease in mitochondrial respiration and total ATP levels upon exposure to FSH. These energetic changes were accompanied by greater ECAR and elevated lactate levels, consistent with enhanced glycolysis. Thus, in contrast to starvation, where mitochondrial elongation maintains ATP levels, FSH-dependent mitochondrial elongation during COC maturation promotes a metabolic switch toward glycolysis. Importantly, this response is most likely driven by cumulus cells since oocytes cannot directly respond to FSH and have a limited capacity to metabolize glucose^23,47^. Additionally, a very low OCR rate was measured in denuded oocytes compared to intact COCs, reflecting the major contribution of cumulus cells^48^.

The mechanism by which FSH can uncouple mitochondrial elongation and ATP production remains unknown but is likely related to changes in cumulus cells’ physiology and metabolism during IVM, such as their proliferation in response to FSH^45^. Cell proliferation is often accompanied by mitochondrial elongation and increased glycolysis, leading to lactate excretion^49^. FSH is already known as a cumulus cell metabolic regulator^23,47,50^. It has been observed that FSH stimulates serum-derived glycogen consumption in bovine COCs during IVM^30^. In mouse and cow COCs, FSH induces high glucose consumption as well as elevated lactate production^51,52^. In ovarian cancer cells, FSH stimulates glycolysis by promoting pyruvate kinase isozyme type M2, which catalyzes the final reaction in glycolysis^53^. In contrast to cumulus cells, oocytes use the pyruvate as an energy source transferred by cumulus cells via gap junctions for ATP synthesis by oocyte mitochondria^47^. This metabolic coordination promotes oocyte maturation^54^.

Oocyte mitochondria also undergo dynamic changes in morphology, number, and distribution during maturation depending on energy demands. Mitochondria appear as rounded forms with few cristae in immature oocytes but form voluminous aggregates upon maturation^55^. The number of mitochondria and DNA copies also increases during maturation, reflecting oocyte quality^56–58^. Mitochondria are redistributed within oocytes, going from a peripheral localization at the immature stage to a diffused appearance after maturation, the latter being associated with functional follicular communication near gap junctions^58,59^. Our results show that changes in mitochondrial structure also occur in cumulus cells, changes that include both mitochondrial length and cristae structure. The latter is significant as changes in the number and structure of mitochondrial cristae, which are key sites of mitochondrial oxidative phosphorylation, regulate organelle ATP synthesis^60^. Therefore, our results, showing changes in mitochondrial cristae number in cumulus cells during IVM, support the changes in OCR that also occur in these cells. As cumulus cells’ functions change during IVM toward the synthesis of hyaluronic acid (for the extracellular matrix) and steroid synthesis, the diminution in mitochondrial ATP synthesis is consistent with the cells’ physiology.

Our study shows that FSH regulates mitochondrial dynamics and activity in cumulus cells. This is consistent with previous studies identifying mitochondria as targets of FSH regulation^61,62^. In bovine oocytes, mitochondrial distribution and ATP synthesis are modulated in response to FSH^63^, while FSH stimulates mitochondrial biogenesis and reduces mitochondrial mitophagy under oxidative stress conditions in porcine granulosa cells^64,65^. Furthermore, it has been demonstrated that FSH attenuates mitochondrial damage by inhibiting mitophagy induced by oxidative stress during aging in mouse granulosa cells^66^. Interestingly, FSH regulates the expression of mitochondrial apoptotic genes in cumulus cells, reduces apoptosis, and improves oocyte developmental competence^67^. Thus, the literature fully supports the FSH regulation of mitochondria in porcine cumulus cells observed in this study.

Finally, we observed that conditions known to produce high cAMP concentration in the pre-IVM culture step^40^ prevent mitochondrial fragmentation after 24h. These observations are consistent with the known role of elevated cAMP concentrations in inhibiting oocyte meiotic resumption, even in the presence of FSH^24,25,38^. Several IVM systems containing FSH were developed in recent years by investigating the effects of a surge in cAMP levels in culture media on oocyte developmental competence^5,24^. The culture of COCs with elevated cAMP levels during the first period of in vitro culture (also known as a pre-IVM culture step) before adding FSH leads to higher rates of oocyte maturation and embryonic development^24,41^. Furthermore, pre-IVM maturation with forskolin + IBMX (to induce a surge in cAMP levels^40^) increases oocyte-cumulus cell gap-junctional communication^41^. This approach improved oocyte developmental capacity and blastocyst quality compared to standard IVM conditions^41^. Importantly, increasing oocyte-cumulus cell communication with high cAMP levels is beneficial for the transfer of nutrients and energy substrates to the oocyte^68^. Based on these previous studies, our results demonstrating a delay of the FSH-mediated mitochondrial response by high cAMP levels during the pre-IVM culture step support a beneficial effect on oocyte, contributing to its maturation and development. However, it is still unclear how a surge in cAMP levels during the pre-IVM culture step delays the long-term FSH-mediated mitochondrial response, which must be assessed in future studies.

According to our results, FSH induces different effects on mitochondrial structure, a short-term effect where mitochondria elongation is measured followed by fragmentation as a long-term effect after 24 h of IVM. Interestingly, the elongation was accompanied by a decrease in mitochondrial activity and a switch to glycolysis. When using a pre-IVM culture step with high levels of cAMP, the long-term effect of FSH was inhibited. These findings reveal that changes in mitochondrial morphology may occur rapidly within minutes but also in the long term, and several factors may be involved.

In conclusion, understanding the effects of FSH on mitochondrial responses in cumulus cells and COC metabolism during IVM is of great interest in the study of follicular physiology and oocyte developmental competence. Using FSH as a modulator of the mitochondrial response and COC metabolism draws attention to its role during IVM and brings new considerations to improve oocyte quality, particularly in combination with high cAMP levels. This approach may have implications for enhancing artificial reproductive technologies.

## Methods

Unless otherwise stated, all chemicals were purchased from Sigma Chemical Company (St. Louis, MO, USA).

### Animals

Live animals were not involved in the study.

### Collection of biological material

Ovaries from pre-pubertal gilts were collected according to a published protocol^69^. Briefly, ovaries were recovered from a local slaughterhouse and placed in saline solution (0.9% NaCl) containing antibiotics and antimycotics (100,000 IU/L penicillin G, 100 mg/L streptomycin, 250 μg/L amphotericin B) and maintained at 37 °C. In the laboratory, the ovaries were rinsed in saline solution, and antral follicles (3 to 6 mm) were punctured using an 18-gauge needle attached to a 10 mL syringe to aspirate follicular cells and follicular fluid. COCs were recovered, washed with HEPES-buffered Tyrode medium (0.01% (w/v) polyvinyl alcohol (PVA-HEPES))^70^ and selected according to criteria described previously^71^. The cells were used immediately or cultured for various periods of time.

### IVM of cumulus-oocyte complexes

Porcine COCs were in vitro matured for different periods of time (0, 4, 24, and 48 h) in North Carolina State University-23 (NCSU-23) medium without bovine serum albumin^72^. The IVM culture medium contained 25 µM 2-mercaptoethanol, 0.1 mg/ml cysteine, 10% (v/v) filtered porcine follicular fluid (PFF), and 0.01 µg/ml recombinant human FSH α/β (R&D Systems, Minneapolis, MN)^31,72^. For each IVM period, ten COCs were incubated in 500 µL of culture medium in 4-well assay plates. Other selected COCs were matured as described above in the presence or absence of FSH for 2 and 24 h to investigate the effects of FSH on mitochondria during IVM.

### Pre-IVM culture step using phosphodiesterase inhibitors

COCs were also cultured in the IVM medium containing FSH with or without PDE inhibitors for 2 and 24 h to block cAMP degradation. We used IBMX (500 µM), a broad-spectrum PDE inhibitor, and PF-04957325 (300 nM, MedChem Express), which is a specific inhibitor of the PDE8 family^73^.

### TMRM staining of active mitochondria

After each culture time, active mitochondria in COCs (10 COCs for each culture period, n = 3) were labeled using TMRM (ThermoFisher Scientific, Waltham, MA) at a final concentration of 150 nM for 30 min at 37.5°C^27^. Once stained, COCs were then washed 3 times with PBS. The North Carolina State University-23 (NCSU-23) medium without bovine serum albumin was used for the final wash and in which COCs were mounted on a glass slide (to ensure proper cell activities for live imaging) using Grace Bio-Labs 200 SecureSeal imaging spacers as described previously^26^. The slides were kept at 37.5 °C during imaging. The second layer of cumulus cells was observed with a confocal live-cell LSM700 microscope, and images were taken using ZEN capture with the 63X objective (laser fluorescence excitation at 555 nm and emission > 560 nm). The mitochondrial phenotype was determined for each IVM period by manual evaluation of the organelle morphology in cumulus cells (from each COC) as very long, long, intermediate, and short (depending on mitochondrial length). When COCs were exposed to FSH or FSH combined with PDE inhibitors for 2 or 24 h, active mitochondria were labeled with TMRM after each experimental condition, and cells were observed live with a confocal microscope, as explained above.

### TMRM confocal image analysis

The Momito algorithm was used to measure the mitochondrial length distribution and connectivity in cumulus cells for each experimental condition, as previously described^28^. First, TMRM confocal images of COCs were converted to binary images. Then, the mitochondrial network of each image was separated into several clusters and was analyzed by the structure interpreter. The information about each cluster structure was then used to provide the length distribution probability of the whole COC image. In addition, EMD values were calculated by the R software (package emdist) using mitochondrial length distributions for each IVM period obtained by Momito, providing information about the distance between two distributions^28^. EMD values were first calculated by comparing mitochondrial distributions for each individual experiment to the average of the three individual experiments that were performed. Calculating these values within an experimental condition measures the experimental variation for each IVM period while comparing the individual distributions for a condition to the average of another condition (for example, 3 individual condition A to the average of condition B and 3 individual condition B to the average of condition A) gives the overall distribution difference between the two conditions. Statistical significance was calculated by one-way ANOVA followed by Tukey’s multiple comparison post hoc test to identify individual differences between EMD means (*p* < 0.05). TMRM confocal images of COCs were also analyzed by the Fiji^74^ software to measure an aspect ratio between mitochondrial length and width during IVM, as previously described^75^. This measure provided information about mitochondrial shape.

### SEM analysis of cumulus cell mitochondrial cristae during IVM

According to an adapted, published protocol, COCs were prepared immediately following IVM for SEM^31^. Briefly, for each IVM period, two hundred COCs were fixed in acrolein solution (3.5%) for 30 min at room temperature. Cells were then washed twice with PBS and pelleted by quick centrifugation. A solid sample mold was then made by mixing cells with 4% agarose to prepare 50 μm sections with a vibratome (Leica VT1000S, Leica Biosystems, Concord, Ont.). Sections were post-fixed 1 h in a 3% potassium ferrocyanide solution combined with an equal volume of 4% aqueous osmium tetroxide, and they were washed five times with ddH_2_O for 3 min each. Tissues were then placed in 0.22 µm Millipore-filtered thiocarbohydrazide solution for 20 min at room temperature and were rinsed again four times with ddH_2_O. Samples were then post-fixed a second time in osmium tetroxide solution (2%) for 30 min at room temperature. The sections were dehydrated in sequential alcohol baths and propylene oxide before being transferred to Durcupan resin between two ACLAR sheets and placed in the oven at 55 °C for 3 days. Ultrathin Sects. (70 nm) were prepared from the regions of interest of the 50 μm sections using a Leica UC7 ultramicrotome. A Zeiss Crossbeam 540 SEM was used to acquire cumulus cell images. For each IVM period, the mitochondrial length was measured by the Fiji software, and cristae structures were manually quantified from 16 cumulus cells of the same COC.

### Extracellular flux analysis

To assess the bioenergetics status of COCs cultured in the presence or absence of FSH, the OCR and the ECAR were measured in vitro in real-time during 6 h with a Seahorse XF HS Mini Analyzer (Agilent/Seahorse Bioscience, North Billerica, MA, USA)y^48,76^. On the day of the assay, all reagents were adjusted to pH 7.4. Following COCs’ recovery, cells were incubated in a Seahorse XFp Cell Culture miniplate (eight wells, 20 COCs/well in triplicate, n = 4) in 175 µl of sterile XF assay buffer (Dulbecco’s Modified Eagle Medium [DMEM; Agilent/Seahorse Bioscience, North Billerica, MA, USA]). The culture medium was supplemented with 10 mM D-glucose, 1 mM Pyruvate, and 2 mM L-glutamine. The miniplate was centrifuged for 3 min at 300 g for cell adherence and incubated for 45 min in an oven at 37 °C for equilibration before the run. The analysis protocol started with an equilibration period of 12 min followed by three measurement cycles (3 min mixing + 5 min waiting + 3 min reading). Then, FSH (0.01 µg/ml) was injected through the injection port, followed by 30 measurement cycles. The OCR and ECAR rates were automatically calculated by the Wave 3.0 software (Agilent/Seahorse Bioscience, North Billerica, MA, USA). To investigate the effects of high cAMP pre-IVM culture step on the response of the COC metabolism to FSH, additional independent experiments (n = 4) were performed to measure the extracellular flux in the presence of FSH ± PDE inhibitors (IBMX 500 mM + PF-04957325 300 nM) or Forskolin (FSK, an adenylyl cyclase stimulator, 100 µM). After three measurement cycles (3 min mixing + 5 min waiting + 3 min reading), FSH ± PDE inhibitors or FSK were injected simultaneously, and the OCR and ECAR were measured in real-time for 30 cycles. After the experiments, the plates were frozen at − 80°C. After thawing, deoxyribonucleic acid (DNA) was quantified using the CyQUANT™ Cell Proliferation Assay (Invitrogen) for data normalization. Fluorescence was read with the Victor2 1420 MultiLabel Counter plate reader (Perkin Elmer Life Sciences, Waltham, MA, USA) and Wallac 1420 software (PerkinElmer, Waltham, MA, USA). The normalization values were calculated from the fluorescence measurements and applied to the metabolic values.

### Mito stress test

The effects of 2 h FSH culture on mitochondrial respiration were also assessed with a Seahorse XF HS Mini Analyzer in the presence of mitochondrial drugs. COCs were cultured for 2 h with or without FSH in Seahorse XFp Cell Culture miniplates (20 COCs/well in triplicate, n = 5) in 175 µl of sterile XF assay buffer (as detailed above) at 37 °C. The miniplate was then centrifuged for 3 min at 300 g for cell adherence and put in the extracellular flux analyzer for OCR and ECAR measurements over 2.5 h. After four measurement cycles (as described above), mitochondrial drugs were loaded through the four injection ports of the cartridge at the specific times indicated in Fig. 4A. A mitochondrial stress test was performed by sequentially injecting the following drugs: oligo (ATP synthase inhibitor, 1 µM), Carbonyl cyanide p-trifluoro-methoxyphenyl hydrazone (FCCP, mitochondrial uncoupler, 1 µM), and rotenone + antimycin A (Rot + AA, complex I and III inhibitors, 0.5 µM each). The OCR and ECAR were measured in pmol/min and mpH/min, respectively, and were normalized by DNA concentration, as described above.

### ATP and lactate assays

A luminescent cell viability assay (CellTiter-Glo® 2.0 assay, Promega) and a bioluminescent assay (Lactate-Glo™ Assay, Promega) were used to measure ATP synthesis and L-lactate production by porcine COCs after 2 h culture in the presence and absence of FSH (0.01 µg/ml) according to the manufacturer’s instructions. Cells (20 COCs/well in duplicate, n = 3) were incubated in an equal volume (100 µl) of NCSU-23 culture medium for 2 h at 37 °C in 96-well assay plates. After 1 h of culture, oligo (10 µM) was added to block ATP synthase and to estimate the mitochondria-dependent ATP production. After a 2 h incubation, 50 µl of the medium was removed (kept for lactate assay), and 50 µl of cell-titer glow ATP substrate (Promega- # G9241) was added to each well to measure the ATP content of the COCs. The luminescence intensity in each well, directly proportional to the number of viable cells, was measured using the FluoStar Omega (BMG LABTECH) plate reader. L-lactate production by the same COC was measured in the removed 50 µl of culture medium, and the luminescent signal was proportional to the concentration of L-lactate present in the medium. The results were normalized by COC number in each well for both the ATP and lactate measurements.

## Statistical analysis

Statistical analysis was performed using GraphPad Prism 8.0.1 for MacOS (GraphPad Software Inc., San Diego, CA). All means are presented with their corresponding SEM. Statistical significance was assessed by Student’s t-test or one-way ANOVA, followed by Tukey’s or Bonferroni’s multiple comparison post hoc test to identify individual differences between means. For statistical analysis of experiments testing the effects of two independent factors, we used two-way ANOVA to compare the mean difference between groups followed by Tukey’s or Bonferroni’s multiple comparison post hoc test. Data were considered statistically significant when *p* < 0.05.

### Supplementary Information

Supplementary Figure 1.

## Data Availability

All data generated during this study are included in this published article.
