# Supplementary material for: The follicle-stimulating hormone triggers rapid changes in mitochondrial structure and function in porcine cumulus cells
Source: Sci Rep. 2024 Jan 3;14:436. doi: 10.1038/s41598-023-50586-3 (PMC10764925; doi:10.1038/s41598-023-50586-3)
Supplement: Supplementary file 1 — Supplementary Figure 1. [file 41598_2023_50586_MOESM1_ESM.pdf]

Supplemental data

The follicle-stimulating hormone triggers rapid changes in mitochondrial structure and function in porcine cumulus cells

Amel Lounas[1], Yann Breton[2], Ariane Lebrun[1], Isabelle Laflamme[1], Nathalie Vernoux[3], Julie Savage[3], Marie-Ève Tremblay[3,4], Martin Pelletier[2], Marc Germain[5] & François J. Richard*[1]

[1]Centre de recherche en reproduction, développement et santé intergénérationnelle (CRDSI), Département des sciences animales, Faculté des Sciences de l'agriculture et de l'alimentation, Université Laval, Québec, Québec, G1V 0A6, Canada.

[2]Centre de recherche du CHU de Québec-Université Laval, Axe Maladies infectieuses et immunitaires, Département de microbiologie-infectiologie et d'immunologie, Faculté de médecine, Université Laval, Québec, Québec, G1V4G2, Canada

[3]Centre de recherche du CHU de Québec-Université Laval, Axe Neurosciences, Département de médecine moléculaire, Université Laval, Québec, Québec, G1V 4G2 Canada

[4]Division of Medical Sciences, University of Victoria, Victoria BC, V8W 2Y2 Canada

[5]Département de biologie médicale, Université du Québec à Trois-Rivières, Québec, G8Z 4M3, Canada.

A

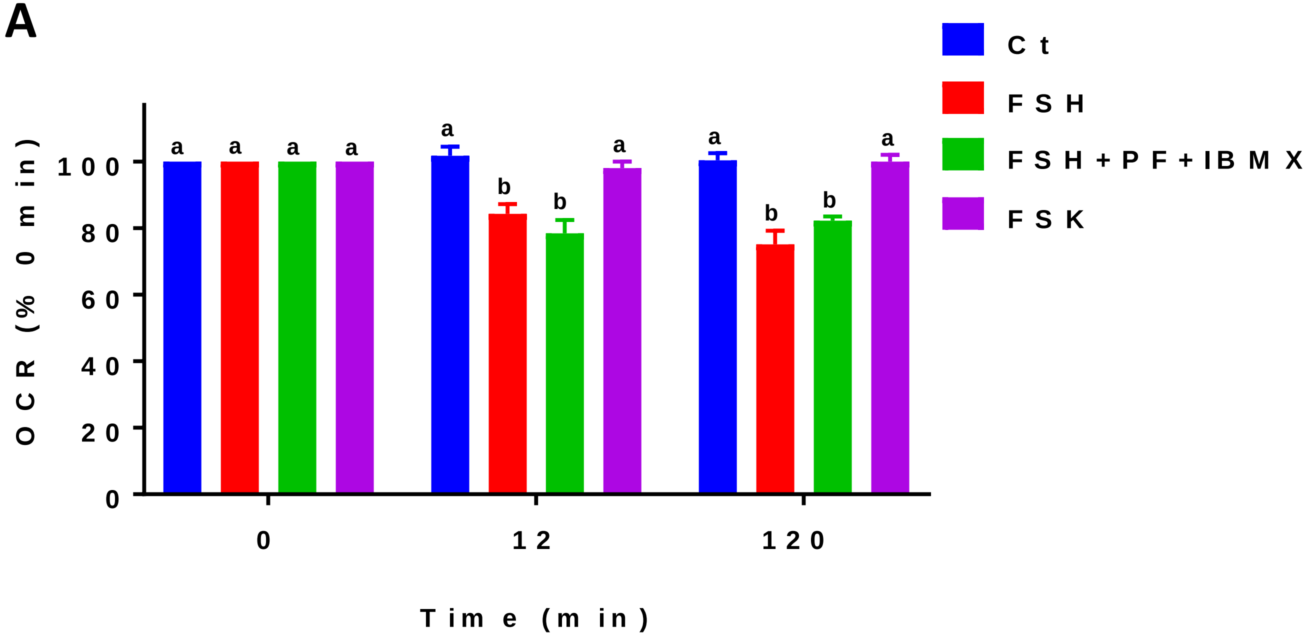

B

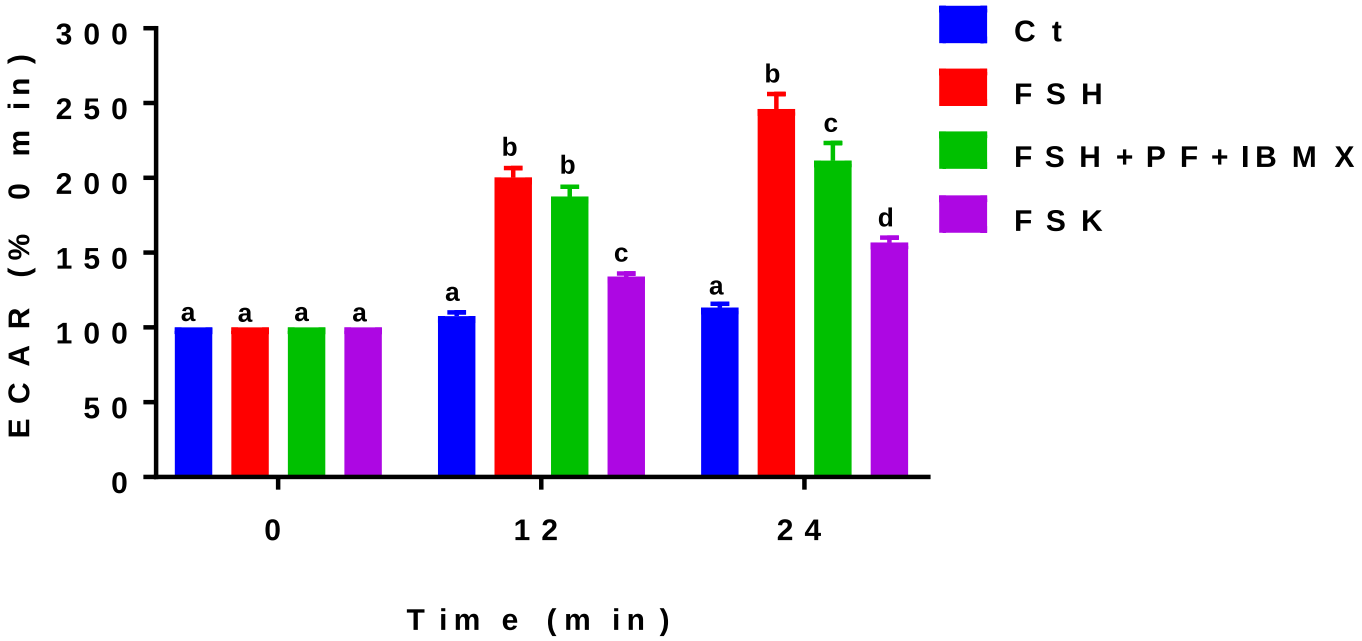

Supplementary figure 1: Effect of pre-IVM culture step with high cAMP levels on FSH-mediated mitochondrial response in COCs. **(A)** Changes in OCR rate percentage of COCs exposed to FSH, FSH+ PDE inhibitors, and forskolin (FSK) compared to control (Ct) for different culture periods. **(B)** Changes in ECAR rate percentage of COCs exposed to FSH, FSH+ PDE inhibitors, and FSK compared to control (Ct) for different culture periods. All data are expressed as the average of four independent experiments ± SEM (20 COCs by well in duplicate). Different letters indicate statistically significant differences ($p < 0.05$).
